# Individual headless compression screws fixed with three-dimensional image processing technology improves fusion rates of isolated talonavicular arthrodesis

**DOI:** 10.1186/s13018-017-0516-0

**Published:** 2017-01-23

**Authors:** Mei-ming Xie, Kang Xia, Hong-xin Zhang, Hong-hui Cao, Zhi-jin Yang, Hai-feng Cui, Shang Gao, Kang-lai Tang

**Affiliations:** Department of Orthopaedic Surgery, Southwest Hospital, The Third Military Medical University, Gaotanyan Str. 30, Chongqing city, 400038 People’s Republic of China

**Keywords:** Individualized preoperative planning, 3D reconstruction, Talonavicular, Arthrodesis, Headless compression screws

## Abstract

**Background:**

Screw fixation is a typical technique for isolated talonavicular arthrodesis (TNA), however, no consensus has been reached on how to select most suitable inserted position and direction. The study aimed to present a new fixation technique and to evaluate the clinical outcome of individual headless compression screws (HCSs) applied with three-dimensional (3D) image processing technology to isolated TNA.

**Methods:**

From 2007 to 2014, 69 patients underwent isolated TNA by using double Acutrak HCSs. The preoperative three-dimensional (3D) insertion model of double HCSs was applied by Mimics, Catia, and SolidWorks reconstruction software. One HCS oriented antegradely from the edge of dorsal navicular tail where intersected interspace between the first and the second cuneiform into the talus body along the talus axis, and the other one paralleled the first screw oriented from the dorsal-medial navicular where intersected at the medial plane of the first cuneiform. The anteroposterior and lateral X-ray examinations certified that the double HCSs were placed along the longitudinal axis of the talus. Postoperative assessment included the American Orthopaedic Foot & Ankle Society hindfoot (AOFAS), the visual analogue scale (VAS) score, satisfaction score, imaging assessments, and complications.

**Results:**

At the mean 44-months follow-up, all patients exhibited good articular congruity and solid bone fusion at an average of 11.26 ± 0.85 weeks (range, 10 ~ 13 weeks) without screw loosening, shifting, or breakage. The overall fusion rates were 100%. The average AOFAS score increased from 46.62 ± 4.6 (range, 37 ~ 56) preoperatively to 74.77 ± 5.4 (range, 64–88) at the final follow-up (95% CI: −30.86 ~ −27.34; *p* < 0.001). The mean VAS score decreased from 7.01 ± 1.2 (range, 4 ~ 9) to 1.93 ± 1.3 (range, 0 ~ 4) (95% CI: 4.69 ~ 5.48; *p* < 0.001). One cases (1.45%) and three cases (4.35%) experienced wound infection and adjacent arthritis respectively. The postoperative satisfaction score including pain relief, activities of daily living, and return to recreational activities were good to excellent in 62 (89.9%) cases.

**Conclusions:**

Individual 3D reconstruction of HCSs insertion model can be designed with three-dimensional image processing technology in TNA. The technology is safe, effective, and reliable to isolated TNA method with high bone fusion rates, low incidences of complications.

## Background

Isolated talonavicular arthrodesis (TNA) is effective to many end-stage talonavicular (TN) arthropathies when conservative treatment is ineffective. Some previous studies reported that the nonunion rates of TNA were 3.8 ~ 11% [1–5]. In most cases, the nonunion rates arise from hardware loosening with loss of interface compression and stability function during the healing process [2, 4, 6–8]. The implants mainly include staples, plates, and screws. Staples and plates are both eccentric fixation with potential disadvantages of extensive soft tissue dissection and impaired blood supply [4, 5, 9]. Screws are most commonly used to fuse TN without above drawback [3, 5, 10, 11]. Different screw designs affect compressive force and fusion rates. Headless compression screws (HCSs) with a variable pitch design generate a maximum compressive force and excellent clinical results [12–14]. Our study group [15] has demonstrated that tibiotalocalcaneal arthrodesis with HCSs was minimally invasive with high fusion rates, less complications.

Nevertheless, because of the narrow talus neck and irregular anatomical structure of talus body, the most preferred screws insertion model is still unclear to TNA. The preoperative and intraoperative position and direction of screws insertion is a key and difficult factor to achieve solid compression and high fusion rates. Most orthopedists always rely on X-ray film or CT to decide the screws insertion model. The accurate screws fixation model cannot be designed through two-dimensional imaging data. Therefore, preoperative screws fixation planning will depend on three-dimensional (3D) tomography reconstruction and processing technology. Xuyi W and Kim YS et al. [16, 17] successfully applied 3D reconstruction technology to design individual operation methods. This technology can mimic the surgery operation and finish preoperative design. The hypothesis of this study was that individual HCSs insertion applied by 3D reconstruction technology to TNA would show preferred fusion rates of TNA comparing with previous studies.

The aim of this study was to reconstruct individual 3D TN joint and double HCSs insertion model from the navicular tail into the talus body through the talus axis and evaluate the clinical safety and effectiveness to TNA.

## Methods

### Clinical data

From 2007 to 2014, 78 consecutive patients were performed with TNA by a senior author (K-lT) with extensive foot surgery experience more than 12 years. This was a single-center and single-surgeon case series. All data were collected and evaluated by independent research staff. This study was approved by the Ethics Committee of the Southwest Hospital Affiliated with the Third Military Medical University (No.ECFAH2006011). All enrolled patients were invited to participate in the hospital registry and provided informed consent. Nine patients were excluded from the initial group of candidate cases in the study cohort of cases with end-stage TN arthropathy. The reason of excluded patients was that they lived too far away to take part in regular follow-up or declined to participate in the registry.

Inclusion criteria included: (1) lack of efficacy to conservative treatments including oral drugs and physical rehabilitation for 6 months at least; (2) severe TN pain, instability, no adjacent joint arthritis from symptoms, and cartilage lesions on MRI; (3) follow-up for 2 years at least.

Exclusion criteria included: (1) age less than 18 years; (2) active deep-seated infection, osteomyelitis; (3) no tolerated anesthesia and surgery because of severe cardiovascular, respiratory, or other medical diseases; (4) patients who had undergone previous surgery of the TN; (5) neuromuscular disease, Charcot arthropathy causing foot imbalance; (6) local muscle strength reduction or muscular atrophy; (7) autogenous or allogenic bone transplantation during the surgeries; (8) severe osteoporosis; (9) hindfoot deformity requiring other joint fusion besides TN.

### Preoperative 3D assembly

**Fig. 1:**
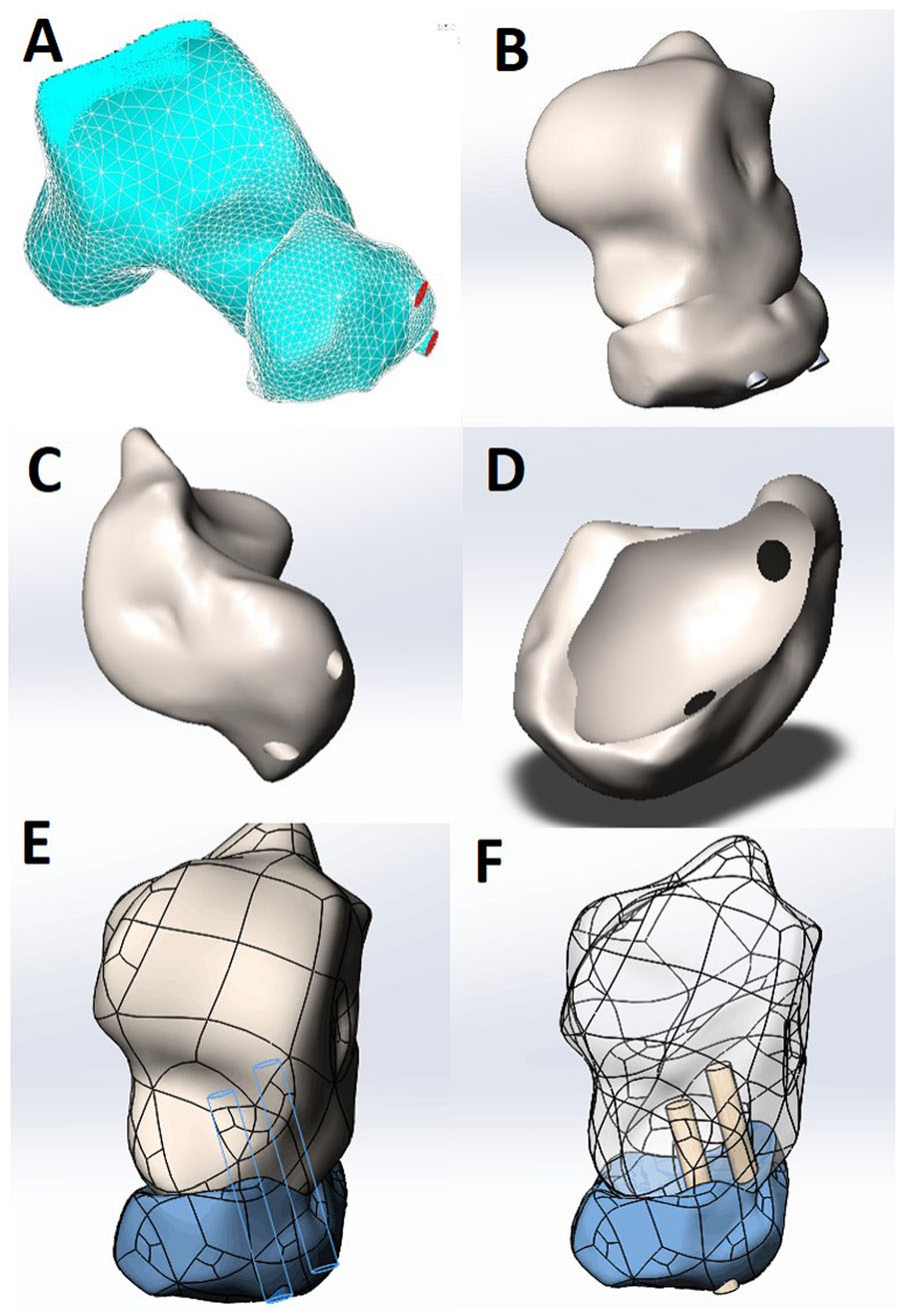
The three-dimensional model of the talus and navicular was reconstructed (**a**, **b**). The segmented surface elements were generated (**c**, **d**). The screws fixation model of TNA was established (**e**, **f**)

CT scans from the tibial plafond to planta pedis were obtained with Siemens Somatom 64-channel scanning (Munich, Germany) under 120 kV, 300 mAs. The slice thickness of talus and navicular CT scanning was 1 mm. The patient was placed in the supine position with the ankle dorsiflexion to neutral position. All CT slice data were transferred to digital imaging in Digital Imaging and Communications in Medicine (DICOM) format (Rosslyn, USA). The bone threshold was 226–1776 HU. The skeletal parts of talus and navicular were extracted and filled with cavity. The images were segmented and reconstructed with 3D model of the talus and navicular by Mimics v17.0 (Materialise, Leuven, Belgium) (Fig. 1a, b). Double HCSs were simplified to be 4.3-mm diameter cylinder. All 3D reconstruction model were transformed to STL+ format. The segmented surface elements were generated in Catia v5 (Dassault, France) (Fig. 1c, d). The screws fixation model of TNA was smoothed and established to determine the space position of HCSs insertion by SolidWorks v2012 (Dassault, France) to analyze spatial relations between double screws and TN in the anatomic structures (Fig. 1e, f). We accurately determined the position and direction of double screws in order to guide intraoperative inserted orientation.

### Surgical technique

**Fig. 2:**
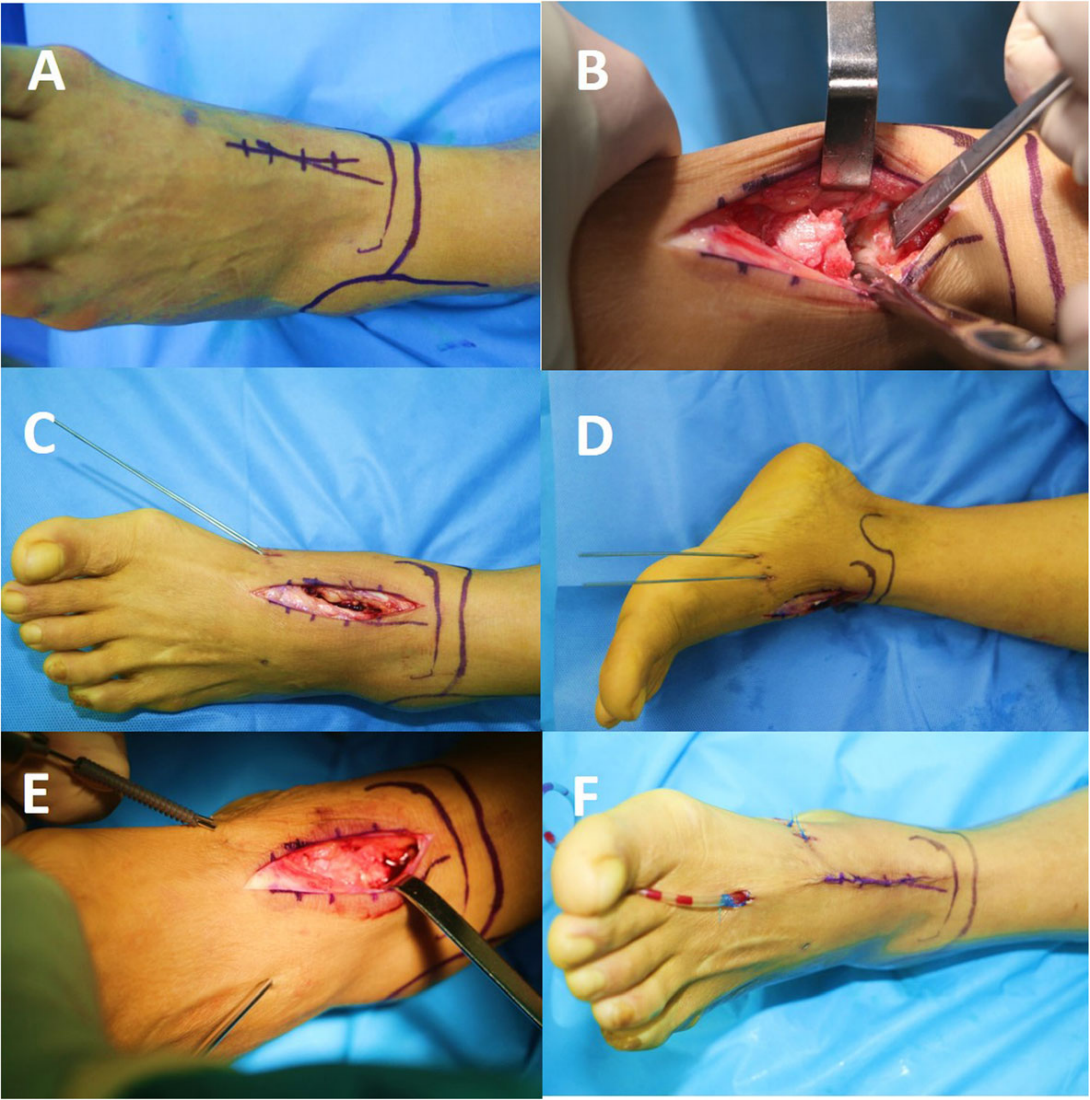
An anteromedial longitudinal incision (**a**) was performed, cartilage was removed (**b**), and the positions of the two guide wires were monitored with AP (**c**) and lateral (**d**) views. Double percutaneous HCSs were placed (**e**). The incision was sutured (**f**)

The patient was placed in the supine position, and a femoral pneumatic tourniquet was inflated. The TN joint was exposed by an approximately 3 cm dorsomedial incision between the tibialis anterior and the posterior tibialis tendon (Fig. 2a). All cartilage was removed using curette and osteotome until bleeding subchondral bone was visualized (Fig. 2b). Following adequate preparation of the bone matching surface, the foot and ankle was placed in neutral flexion to preserve the natural contour, and the interfragmentary gap was disappeared.

**Fig. 3:**
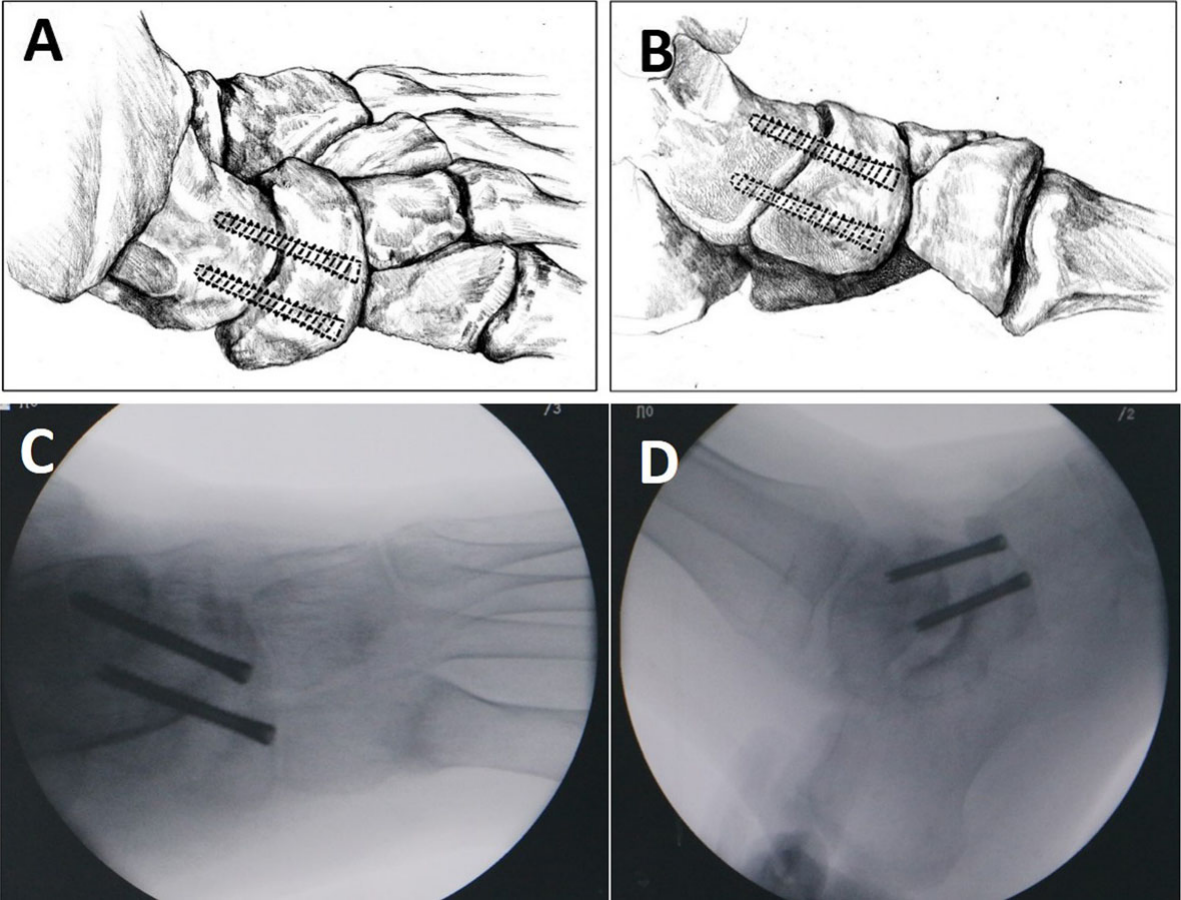
The AP (**a**, **c**) and lateral (**b**, **d**) views showed double HCSs located at equal division points of talus neck width and height

One 1.5-mm guide pin was placed from the edge of dorsal navicular tail where intersected interspace between the first and the second cuneiform antegradely using a protractor. It was drilled into the talus body along the talus axis according to preoperative designed orientation by fluoroscopic image intensification. The other 1.5-mm guide pin paralleled the first one and oriented from the dorsal-medial navicular where intersected at the medial plane of the first cuneiform (Fig. 2c, d). The lateral and anteroposterior (AP) fluoroscopy checked the position of double guide pins where it was within the talar neck and body. A 2.0-mm guide pin temporarily fixed the TN from the outside, and the TN interspace completely disappeared. Thereafter, double percutaneous HCSs (4.3-mm in diameter, 40 mm to 60 mm in length, 1.5-mm in pitch, 0.2-mm increased pre ring, Acumed Ltd., Hillsboro, OR, USA) were placed through the TN interface along the double guide pins (Fig. 2e). All guide pins were removed. The AP and lateral view showed that double HCSs tried to parallel into the lateral talus body located in the equal division point of the talus width and height (Fig. 3). The heads of double HCSs were buried under the cartilage by the osteosynthesis lag technique to avoid screw prominence. The TN interspace disappeared after tightening the double HCSs checked by fluoroscopy. The incision was sutured with a negative pressure drainage tube (Fig. 2f).

### Postoperative management and data collection

The negative pressure drainage tube was removed after 24 h. The partial weight bearing with aircast for 4 weeks and then full weight-bearing with aircast for a remaining 6 to 8 weeks if continuous and solid callus was formed on the TN as shown by X-ray examination. All data were paired before surgery and at the last follow-up.

All patients were assessed by clinical examinations: (1) imaging assessments: the standing AP and lateral total foot X-rays to evaluate the radiological fusion that callus passed through the TN interspace or if the interspace was disappeared; (2) functional outcome assessments: the American Orthopaedic Foot & Ankle Society hindfoot score (AOFAS), the visual analogue scale (VAS) score to evaluate postoperative recovery [1–3, 9, 10]; (3) postoperative complication assessments: the incidence rates of complications to evaluate the side effects, the satisfaction scores [15] regarding pain relief, activities of daily living, and return to recreational activities.

### Statistical analysis

Continuous data were descripted as means ± SD. The paired-samples *t* test was performed for statistical analysis to compare the preoperative and postoperative AOFAS, VAS score using the Statistical Package for Social Sciences, version 13.0 (SPSS INC., Chicago, IL, USA). All tests were two-tailed, and *p* < 0.05 suggested a statistically significant difference. The 95% confidence interval of the difference (CI) was recorded as the paired difference.

## Results

### Demographic data

The mean follow-up duration was 44 months (range, 24 ~ 115 months). With a mean of 54.28 ± 9.6 years (range, 34 ~ 75 years), 39 patients were men and 30 patients were women. The duration of disease was 14.48 ± 6.1 months ((range, 6 ~ 28 months). Twenty-eight patients suffered from isolated TNA on the left side and 41 on the right. Twenty-four (34.8%) patients had a preoperative diagnosis of posttraumatic arthritis; fifteen (21.7%), primary osteoarthritis; five (7.2%), inflammatory osteoarthritis; eight (11.6%), rheumatoid arthritis; nine (13.0%), adult-acquired flatfoot; six (8.7%), juvenile pas calcaneal valgus and two (3.0%), posterior tibial tendon insufficiency (Table 1).

**Table 1:** Patient demographic data

Male/female	39/30
Age at op. (years)	54.28 ± 9.6
Duration(ms)	14.48 ± 6.1
Left/right (no.)	28/41
Diagnosis	
Posttraumatic arthritis	24(34.8%)
Primary osteoarthritis	15(21.7%)
Inflammatory osteoarthritis	5(7.2%)
Rheumatoid arthritis	8(11.6%)
Adult-acquired flatfoot	9(13.0%)
Juvenile pas calcaneal valgus	6(8.7%)
Posterior tibial tendon insufficiency	2(3.0%)

### Imaging assessments

**Fig. 4:**
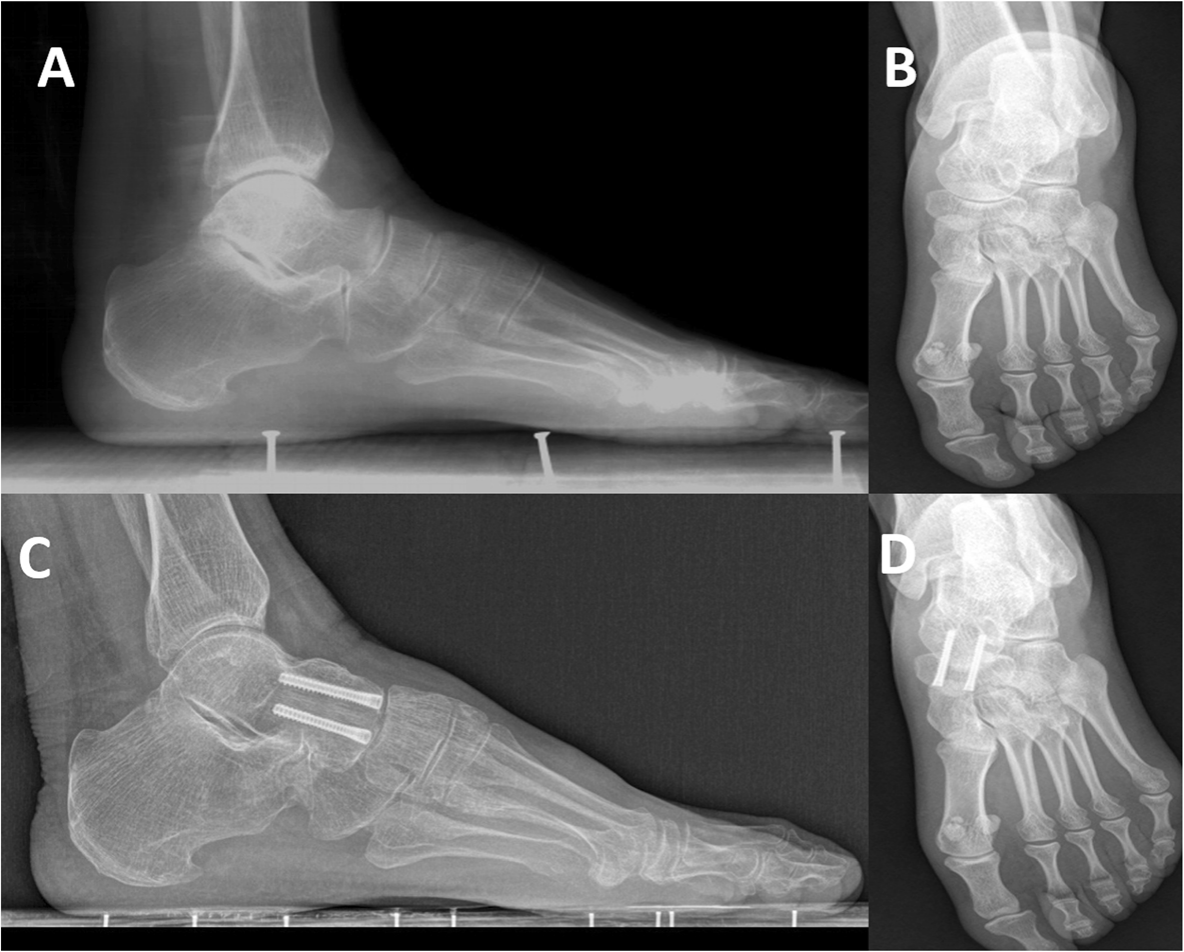
Preoperative (**a**, **b**) and postoperative (**c**, **d**) radiographs to TNA with double HCSs after 4 years

All patients achieved solid bone fusion as controlled by preoperative X-rays radiographs (Fig. 4). The average bone fusion occurred at 11.26 ± 0.85 weeks (range, 10 ~ 13 weeks). The overall fusion rates were 100%.

### Functional outcome assessments

The average AOFAS score improved from an average of 46.62 ± 4.6 (range, 37 ~ 56) preoperatively to 74.77 ± 5.4 (range, 64 ~ 88) at the final follow-up (95% CI: −30.86 ~ −27.34; *p* = 0.000). The VAS score decreased from 7.01 ± 1.3 (range, 4 ~ 9) to 1.93 ± 0.3 (range, 0 ~ 4) (95% CI: 4.69 ~ 5.48; *p* = 0.000) (Table 2).

**Table 2:** AOFAS and VAS score before operation and at the last visit

Scales	Preoperation	The last visit	95% CI	*t* value	*p* value
AOFAS score	46.62 ± 4.6	74.77 ± 5.4	−30.86 ~ −27.34	−31.86	0.000
VAS score	7.01 ± 1.2	1.93 ± 1.3	4.69 ~ 5.48	25.72	0.000

### Complication assessments

No patient required secondary hardware removal surgery for symptomatic metalwork. One patient (1.45%) required antibiotics for wound infection, without return to the operating room. Three patients (4.35%) complained of residual pain because of hindfoot and transverse tarsal arthritis. The postoperative satisfaction scores regarding pain relief, activities of daily living, and return to recreational activities were good to excellent in 62 (89.9%) cases.

## Discussion

Clinical and biomechanical trials have shown that rigid internal fixation in isolated TNA lead to higher rates of union and lower infection rates and earlier ROM [2, 18]. Screw fixation is a traditional and a popular technique to isolated TNA. However, consensus has not reached the most suitable screw insertion model [18]. Over the years, we have enhanced the subtalar and tibiotalocalcaneal stability and arthrodesis rates using double HCSs paralleling through the bone axis [15, 19, 20]. So we attempted to fix TN with double HCSs.

In this study, the postoperative AOFAS score increased significantly, which is similar to the results reported by Lu CK and Shymon et al. [1, 9, 10]. All patients were reported to obtain significant pain relief and improved overall function according to VAS score. Lechler P et al. [4] systematically reviewed that 125 combined cases of isolated TNA have variable results, with rates of complications ranging from 10 to 37.5%; the overall complication rates were 20.8%, including nonunion, adjacent arthritis and hardware-related problems. Barkatali BM and Munoz MA et al. [1–5] reported the nonunion rates of TNA were up to 3.8 ~ 11% and the operation was difficult to obtain satisfactory fusion rates. It is worthy to note that our fusion rates were as high as 100% without any interface screws loosening. Besides, Carranza-Bencano A et al. [10] found that 10 ~ 33% of the patients who underwent TNA developed adjacent joint degeneration. In our study, 1.45% of cases required antibiotics for wound infection, and 4.35% of cases suffered from adjacent arthritis without other complications. Compared the clinical complication rates with traditional screws fixation previously reported, our technique showed better recovery, lower incidence rates of complications, higher fusion rates. The satisfactory clinical results may be associated with these advantages as below.

Firstly, double HCSs can avoid the TN rotation and insert in the ample space. One to three screws have commonly been used to fix TN interface [2, 10]. One screw alone cannot prevent interface rotation. Carranza-Bencano, A et al. [10] applied one screw to insert TN from the posterior part of the talus to the navicular body. This screw insertion is difficult and dangerous because the posterior ankle is rich in blood vessels and nerves. Most surgeons utilized two or three screws inserted from the navicular into the talus body. Jarrell SE, 3rd et al. [18] suggested that compressive force with three screws was greater than with two screws through a biomechanical study. Van den Broek, M and Barkatali, B. M et al. [2, 21] adopt three screws fixation to fuse TN. However, the limited space of the talus and navicular body would make fixation with three screws difficult because of screw collisions and extensive soft tissue exposure. Three screws increase more cost of health care than 2 screws. Hence, we chose double screws fixation model.

In addition, the inserted landmarks were easy to be located and compress the TN interspace as vertically as possible at lower plane. The largest contact forces through interfacial forces provide a higher torque of insertion and pullout strength or failure load [22–24]. The selection of specific entry points usually leads to the determination of different fixation models. Due to the key biomechanics and anatomical features of the navicular, the lower the insertion plane of the screws, the easier it is to vertically compress the TN interspace. So the perfect screws were inserted from the navicular-cuneiform joint, rather than from the dorsal navicular. However, the navicular-cuneiform joint cannot be fixed in operation, so we chose double HCSs to insert form the edge of dorsal navicular tail where intersected interspace between the first and the second cuneiform and from the dorsal-medial navicular where intersected at the medial plane of the first cuneiform.

Furthermore, double HCSs preferably paralleled with the talus axis in the AP and lateral views, to avoid implant loosening due to stress shielding. Yuan C. S et al. [19, 20] found that two HCSs are an ideal screw insertion method in stress distribution and anti-inversion/eversion strength to subtalar arthrodesis. Matsumoto, T et al. [25] demonstrated that double screws fixation produced significantly higher average compression than did diverging double screws fixation. Talus 3D space position should be considered to avoid stress shielding. Screws provide a means of compression across talus axis to maintain stability. The TN joint can be difficult to fuse because of its instability from shear and torsion forces [18]. Double paralleling HCSs can provide continuous and stable pressure from the TN interspace to the talus body through the talus axis.

Finally, to improve the accuracy of screw insertion, a preoperative design to each individual based on the computer aided modeling by Mimics, Catia, and SolidWorks software. The development of computer imaging software enables the individual surgery design. Computational models can further evaluate the model of hindfoot diseases [26]. The anatomy of talus and navicular is irregular geometry. It was difficult to accurately assess the position and direction of double HCSs in order to select the most appropriate placement by preoperative computer design techniques. The 3D spatial structure of double HCSs can be computerized by image processing technique in individual design of fixation methods. The preoperative planed the simulation procedure has been used to some orthopedics surgery [16, 17]. We applied above technology to reconstruction TN alignment and mimic screw insertion model. The preoperative planning can design that double screws paralleled with the longitudinal axis of the talus body in accordance with the talus anatomy feature. The study using 3D computerized tomography reconstruction and image processing technology demonstrated its efficacy for precise evaluation of double screws inserting into irregular talus body.

However, this study has some limitations. First, due to lack of a typical screw fixation group to TNA, a standard control study cannot be designed. Second, we have neglected the influence of the pathogen on the outcome. Furthermore, cadaver specimen and 3D finite element studies will confirm distribution of the interface force. In future research, we will look to design more convenient and effective 3D imaging processing technology to treat with personalized TNA.

## Conclusions

The individual geometric feature of the TNA and HCSs insertion model can be applied by using 3D computerized tomography reconstruction and image processing technology. Based on the reconstruction technology, orthopedists can design individual HCSs insertion surgery planning which is safe, accurate, and adequate to isolated TNA with 100% fusion rates, low rates of complication postoperatively.

## Abbreviations

3D: Three-dimensional
AOFAS: American Orthopaedic Foot & Ankle Society
CI: Confidence interval
HCS: Headless Compression Screws
ROM: Range of motion
TN: Talonavicular
TNA: Talonavicular arthrodesis
VAS: Visual analogue scale
